# A clinical study of inferior mesenteric artery typing in laparoscopic radical resections with left colonic artery preservation of rectal cancer

**DOI:** 10.1186/s12957-022-02762-4

**Published:** 2022-09-12

**Authors:** Jinghao Chen, Meirong Wang, Yuhao Chen, Suying Chen, Jing Xiao, Xiaole Fan, Jushun Yang, Bosheng He

**Affiliations:** 1 Department of Radiology, Affiliated Hospital 2 of Nantong University, No.6 Hai Er Xiang North Road, Nantong, 226001 Jiangsu Province China; 2 Department of Radiology, Affiliated Hospital of Nantong University, Nantong, 226006 Jiangsu Province China; 3 Department of Epidemiology and Medical Statistics, School of Public Health, Nantong University, Nantong, 226019 Jiangsu Province China; 4 Nantong Key Laboratory of Intelligent Medicine Innovation and Transformation, Nantong, 226001 Jiangsu China

**Keywords:** Inferior mesenteric artery, Laparoscopic, Left colic artery, Rectal cancer

## Abstract

**Objectives:**

An investigation of the effects of different types of the inferior mesenteric artery (IMA) on laparoscopic left colic artery (LCA) radical resection of rectal cancer was conducted.

**Methods:**

Clinical data were collected from 92 patients who underwent laparoscopic radical resection of rectal cancer with preservation of the LCA at Nantong University’s Second Affiliated Hospital. All patients underwent full-abdominal dual-energy CT enhancement examination before surgery and 3D post-processing reconstruction of the IMA. Two radiologists with >3 years of experience in abdominal radiology jointly conducted the examination. A total of three types of IMA were identified among the patients: IMA type I (the LCA arising independently from the IMA), type II (LCA and sigmoid colon artery [SA] branching from a common trunk from IMA), and type III (LCA, SA, and superior rectal artery [SRA] branching from the IMA at the same point). The baseline data, pathological results, and intra-operative and post-operative indicators of the groups were analyzed.

**Results:**

The proportions of type I, type II, and type III IMA were 58.70% (54/92), 18.48% (17/92), and 22.82% (21/92), respectively. IMA typing was consistent with the preoperative CT evaluation results. The intra-operative blood loss of type III IMA patients [median (interquartile spacing), M (P25, P75): 52.00 (39.50, 68.50) ml] was higher than that of type I and II IMA patients [35.00 (24.00, 42.00) and 32.00 (25.50, 39.50) ml, respectively] (*P*<0.05). The incidence of anastomotic fistula in type III IMA patients (4 cases, 19.05%) was higher than that in non-type III IMA patients (1 case, 1.41%) (*X*^2^=6.679, *P*=0.010). The incidence of postoperative complications among the three types of IMA was not significantly different (*P*>0.05).

**Conclusions:**

Among rectal cancer patients undergoing laparoscopic LCA preservation, type III IMA patients had more intraoperative bleeding and a higher incidence of postoperative anastomotic fistula. However, this did not increase the risk of overall postoperative complications.

## Highlights

1. DECT three-dimensional reconstruction can accurately evaluate the classification of IMA before operation.

2. The short-term outcome of Dixon surgery with LCA preservation is affected by the type of IMA presented.

## Introduction

Total mesorectal excision (TME) and standardized lymph node dissection are the standard surgical procedures for rectal cancer [1, 2]. At present, the main surgical methods include open surgery, Da Vinci robotic surgery, and laparoscopic surgery. Among these, laparoscopic surgery provides less trauma, lower cost, and shorter recovery time for patients [3, 4].

The inferior mesenteric artery (IMA) supplies blood to the left colon and rectum [5]. During laparoscopic radical resection of rectal cancer, precise ligation of the IMA directly affects the blood supply of the postoperative intestinal anastomosis, thus affecting the prognosis for patients [6]. However, the IMA has a high rate of anatomical variation. In other words, its branch types vary from person to person. Previous studies divided IMA into four types [7]. The length of different types of IMA is different, and the branches of IMA and the course of inferior mesenteric vein (IMV) are also different [8, 9].

At present, the treatment of the IMA in laparoscopic radical resection of rectal cancer is controversial [10–13]. Debate centers on whether to preserve the left colic artery (LCA) and the impact of alternative approaches on patient outcomes. However, the prognosis of patients with different IMA types in laparoscopic radical resection of rectal cancer with LCA reservation has rarely been reported. Hence, this study sought to investigate the intra-operative and postoperative short-term outcomes of different IMA classifications in patients with colorectal cancer undergoing laparoscopic LCA preservation.

## Materials and methods

### Patients

Patients with suspected rectal cancer who were admitted to the Department of Gastrointestinal Surgery and Gastroenterology of the Second Affiliated Hospital of the Nantong University between January 2020 and September 2021 were selected. The ethics committee of the Second Affiliated Hospital of Nantong University approved this study (approval number: 2020YKS024).

The inclusion criteria of this study were as follows: (1) rectal cancer confirmed by colonoscopy or pathology, (2) laparoscopic resection of rectal cancer reserving LCA, (3) distance from the tumor to the anus ≥7 cm, and (4) patients who underwent total abdominal enhancement with dual-energy CT before surgery.

The exclusion criteria of this study were as follows: (1) patients with IMA atherosclerosis and stenosis, (2) previous abdominal surgery, (3) patients with intestinal diseases requiring emergency surgery (e.g., intestinal perforation, necrosis, or obstruction), (4) patients with distant organ metastasis, and (5) the lack of LCA IMA.

We enrolled 271 patients. Ninety-one patients with no rectal cancer were excluded from the study. Among the patients who were diagnosed with rectal cancer, the following were excluded: A total of 16 patients underwent laparoscopic rectal cancer resection without LCA preservation for various reasons, which included IMA atherosclerosis in 12 patients and stenosis in four patients without LCA type IMA. A total of seven patients had a history of abdominal surgery. To understand the postoperative gastrointestinal recovery of patients, the time of first anal discharge was analyzed, and 43 patients with tumors < 7 cm from the anal margin who underwent the Miles surgery were excluded. There were two patients who underwent gastrointestinal emergency surgery. A total of 19 patients had distant metastasis. Finally, one patient had incomplete clinical data.

A total of 92 patients were included in the analysis. In particular, there were 63 male participants aged 45–88 years, with an average age of 67.70±10.14 years, and 29 female patients aged 50–83 years, with an average of 66.76±9.85 years.

### Intestinal preparation and collection parameters of preoperative total abdominal enhanced CT

The patients fasted for 4–8 h and underwent dual-energy CT with Siemens Dual-source CT (Somatom Force, Siemens Healthcare, Forchheim, Germany). The scan parameters and scheme details are presented in Table 1.

**Table 1:** Dual-energy CT parameter acquisition and scanning scheme

Group	Scanning scheme/parameters
Scanned area	Diaphragmatic apex to lower margin of the symphysis pubis
Body position	Supine position
Tube voltage	A ball tube 90 kV/B ball tube SN 150 kV
Tube current	A ball tube 144 mAs/ B ball tube 90 mAs
Linear fusion coefficient	0.5
Pitch	1.0
Rotate speed	0.5 s
Collimation	2×192×0.6 mm
Slice thickness	1 mm
Scanning interval	1 mm
Monitoring technique	Phase III enhancement (automatic tracking technology: when the aorta monitoring threshold reaches 100 HU, the scanning of the artery phase is triggered, the venous phase scanning is performed after 40 s, and the delayed phase scanning is performed 80 s after the completion of the venous phase scanning)
Contrast agent	Iopromide
Contrast injection concentration	370 mgI/ml
Contrast injection dose	1.5 ml/kg
Contrast injection rate	3.5 ml/s
Subsequent normal saline	20~30 ml

### CT image reconstruction and IMA typing judgment

**Fig. 1:**
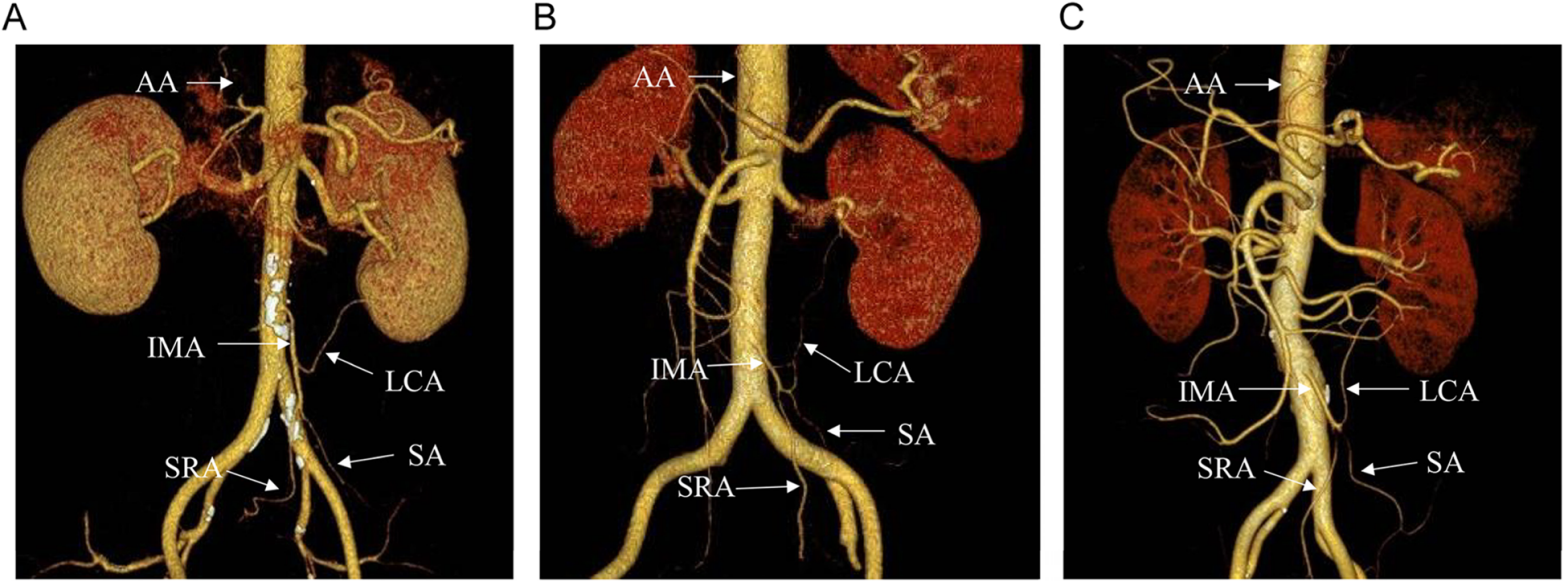
Branching types and distribution of the IMA. **A** Type I IMA, **B** Type II IMA, **C** Type III IMA. IMA inferior mesenteric artery, LCA left colonic artery, SA sigmoid artery, SRA superior rectal artery, AA abdominal aorta

Following scanning, the arterial phase data were transmitted to a Siemens Synovia post-processing workstation. Oblique coronal thin-layer maximum density projection (THIN-MIP) images were obtained through 3D reconstruction of 120 kVp linear fusion images (parallel to the long axis of the IMA, with a layer thickness of 10 mm; the layer spacing was 5 mm). Additionally, volume rendering (VR) images of automatic bone removal were obtained. According to IMA Yada [7], two radiologists with 5 and 10 years of abdominal radiology experience classified the images. The types were as follows: IMA type I (the LCA arising independently from the IMA), type II (LCA and sigmoid colon artery [SA] branching from a common trunk from IMA), and type III (LCA, SA, and superior rectal artery [SRA] branching from the IMA at the same point) (Fig. 1). If opinions of the radiologists were not the same, the conflict was resolved through consultation with a third party.

### Surgical method

Laparoscopic LCA-sparing radical resection of rectal cancer was performed according to the TME standards. Tracheal intubation and general anesthesia were performed. Carbon dioxide was administered to establish pneumoperitoneum. The Toldt fascia alba was cut using an ultrasonic knife to find the IMA root and to avoid damage to the peripheral vascular nerves. The exposed IMA to the LCA was accurately preserved according to the IMA classification. LCA was retained in all three IMA types, and SA and SRA were cut with a Hamolock clamp. Another Hamolock clamp was used to cut off the IMV. Lymph nodes between IMA, LCA, and IMV were dissected. The Gerota fascia was found around the left kidney. The bowel was then dissociated. Tumor resection was performed a 3-cm distal to the tumor. Rectosigmoid anastomosis was performed with a tubular stapler through the anus, and the anastomosis was strengthened by intermittent suture with absorbable suture. The abdominal wound was rinsed to stop the bleeding. Two drainage tubes were inserted, and the puncture holes and incisions were sutured individually.

### Pathological, intraoperative, and postoperative indicators were observed

The pathological indicators were as follows: the pathological stage of the tumor, number of dissected lymph nodes, and positive rate of lymph nodes. The intra-operative observation indexes were as follows: intra-operative time, intra-operative blood loss, postoperative indicators, first postoperative anal discharge time, residence time of the abdominal drainage tube, postoperative hospital stay, and postoperative complications (anastomotic fistula, intestinal obstruction, intestinal ischemia, and abdominal infection).

### Postoperative follow-up methods

The discharged patients were followed up via telephone and outpatient reviews. Plain chest and whole abdominal plain scans were performed for re-examining patients. An enhanced scan was conducted as necessary. Fiber colonoscopy was performed in some patients. Follow-up ended in October 2021, with a median follow-up time of 5.00 months (range: 3.00–8.25).

### Statistical analysis

Statistical analysis was performed using the Statistical Package for the Social Sciences software (SPSS 25.0, IBM corporation).

Continuous variables with normal distribution are expressed as mean ± standard deviation (*X*±*S*). One-way ANOVA was used for the multi-group comparisons. Continuous variables had non-normal distribution. They are represented as median (interquartile spacing) [M (P25, P75)]. The Kruskal-Wallis test was used for comparison between groups, and the Mann-Whitney *U* test was used for comparison between groups. Categorical variables are expressed as percentages using the Pearson chi-square test or the continuity-corrected chi-square test. *P* < 0.05 was statistically significant.

## Results

### IMA typing and baseline data of the patients

Among the 92 patients who underwent laparoscopic LCA-preserving radical rectal cancer resection, the proportion of type I IMA was 58.70% (54/92), that of type II IMA was 18.48% (17/92), and that of type III IMA was 22.82% (21/92). The actual intra-operative exploration of IMA typing was consistent with the preoperative CT assessment (100%). Among patients with different IMA types, sex, age, body mass index, and distance between tumor and anus did not differ significantly. (*P*>0.05). The specific situations are presented in Table 2.

**Table 2:** Baseline data of patients with different IMA classifications

Parameter	Type I IMA (*n*=54)	Type II IMA (*n*=17)	Type III IMA (*n*=21)	*X*^*2*^*/F*	*P*
Sex				0.670	0.967
Male, *n* (%)	37 (68.52)	12 (70.59)	14 (66.67)		
Female, *n* (%)	17 (31.48)	5 (29.41)	7 (33.33)		
Age, mean ± SD (years)	68.63±9.87	68.24±7.65	63.57±11.42	2.052	0.135
Body mass index(kg/m^2^)	24.51±4.03	23.50±3.06	23.06±2.80	1.396	0.253
The distance from the tumor to the anus(cm)	9.31±3.75	9.94±3.60	11.10±3.25	1.863	0.161

### Relationship between different IMA types and the pathological results of patients

The TNM stages of all rectal cancer patients were as follows: stage I: 21 (22.83%), stage II: 33 (35.87%), and stage III: 38 (41.30%). There were no differences in TNM stage, a number of lymph nodes dissected, or positive rate of lymph nodes among the three groups of patients with different IMA types (*P*>0.05). Details are presented in Table 3.

**Table 3:** Pathological data of patients with different IMA types

Pathological indicators	Type I IMA (*n*=54)	Type II IMA (*n*=17)	Type III IMA (*n*=21)	*X*^*2*^*/F*	*P*
TNM, *n* (%)				1.129	0.890
I	13(24.07)	3(17.65)	5(23.81)		
II	18(33.33)	6(35.29)	9(42.86)		
III	23(46.60)	8(47.06)	7(33.33)		
Number of dissected lymph nodes, mean ± SD (*n*)	21.80±4.88	21.35±5.04	22.24±3.51	0.172	0.842
Overall lymph node positive rate, *n* (%)	23(42.59)	8(47.06)	7(33.33)	0.820	0.664

### The relationship between different IMA types and the patients’ intra-operative and postoperative indices

Among patients undergoing laparoscopic radical resection of rectal cancer with LCA reservation, the intra-operative blood loss of type III IMA patients [52.00 (39.50, 68.50) ml] was higher than that of type I [35.00 (24.00, 42.00) ml] and type II [32.00 (25.50, 39.50) ml] patients (*P*<0.05). There was no difference in intra-operative blood loss between patients with type I and type II IMA (*P*>0.05). Further combined with type I and type II IMA patients, the intra-operative blood loss of type III IMA patients [52.00 (39.50, 68.50) ml] was higher than that of non-type III IMA patients [34.00 (24.00, 41.00) ml], (Z = -3.505, *P*<0.001). Although the operative time of type I IMA patients of 145.00 (140.00, 157.50) min was lower than that of type II [170.00 (130.00, 210.00) min] and III [175.00 (140.00, 210.00) min] patients, there was no significant difference in the operation time among the three groups (*P*>0.05) (Tables 4 and 5).

**Table 4:** Intraoperative and postoperative indexes of patients with different IMA types

	Type I IMA(*n*=54)	Type II IMA (*n*=17)	Type III IMA (*n*=21)	*Hc/X*^*2*^	*P*
Intraoperative blood loss, M (P25, P75)(mL)	35.00(24.00, 42.00)	32.00(25.50, 39.50)	52.00(39.50, 68.50)	12.287	0.002
Operation time, M (P25, P75)(min)	145.00(140.00, 157.50)	170.00(130.00, 210.00)	175.00(140.00, 210.00)	3.924	0.141
Abdominal drainage tube retention time, M (P25, P75)(day)	15.00(12.75, 16.00)	16.00(13.50, 18.00)	16.00(15.00, 16.50)	3.224	0.199
Time of first anal discharge, M (P25, P75)(day)	3.00(2.00, 4.00)	4.00(3.00, 4.00)	3.00(2.00, 3.00)	5.394	0.067
Postoperative hospital stay, M (P25, P75)(day)	16.00(14.00, 18.25)	17.00(15.00, 20.00)	17.00(15.00, 17.50)	2.181	0.336
Overall postoperative complications, *n* (%)	4(7.41)	1(5.88)	4(19.05)	2.375	0.308

**Table 5:** The relationship between intraoperative blood loss and anastomotic fistula incidence in type III and non-type III IMA patients

	Type III IMA (*n*=21)	Non-type III IMA (*n*=71)	*Hc/X*^*2*^	*P*
Intraoperative blood loss, M (P25, P75)(ml)	52.00(39.50, 68.50)	34.00(24.00, 41.00)	-3.505	<0.001
Anastomotic stoma fistula, *n* (%)	4(19.05)	1(1.41)	6.679	0.010

The incidence of postoperative anastomotic fistula in type III IMA patients (4 cases, 19.05%) was higher than that in non-type III IMA patients (1 case, 1.41%) (*X*^*2*^=6.679, *P*=0.010). However, in all three groups, postoperative complications were not different (*X*^*2*^=2.375, *P*=0.308). In addition, this study also compared the time of the first postoperative anal discharge, postoperative retention time of the abdominal drainage tube, and postoperative hospital stay in the three groups of IMA patients and found no statistical differences (*P*>0.05), as shown in Tables 4 and 5.

## Discussion

### The advantages of preserving the LCA in laparoscopic radical resection of rectal cancer

In the surgical history of rectal cancer resection, Miles [14] and Moynihan [15] proposed the LCA-preserving low IMA ligation method and the non-LCA-preserving high IMA ligation method successively in 1908. However, since then, the superiority of the LCA-preserving low IMA ligation method has not yet been determined.

In recent years, an increasing number of domestic and foreign scholars [16–19] supported the preservation of the LCA to increase blood supply to the intestinal wall of the postoperative anastomosis. In terms of lymph node dissection and tumor outcome, Maeda [16] reported that low ligation of the IMA and standardized dissection of the D3 lymph node had the same effect as high-ligation lymph node dissection. Luo [17] found that in laparoscopic radical resection of rectal cancer, there was no significant difference in the number of lymph nodes obtained by LCA-preserving low ligation and high ligation, and the 3-year, 5-year, overall, and disease-free survival rates of patients with the two surgical methods were similar. In terms of complications to patients, You [18] found that compared with low ligation, high ligation of the IMA reduced anastomotic intestinal blood supply, increased the incidence of postoperative anastomotic stenosis and anastomotic fistula, and damaged the submesenteric nerve plexus, thereby increasing the incidence of postoperative urinary and reproductive system dysfunction. Zhou [19] found that precise laparoscopic low ligation based on IMA typing could reduce the incidence of overall postoperative complications compared to that with high ligation. Therefore, preservation of the LCA during laparoscopic radical rectal cancer surgery could reduce the incidence of postoperative complications without affecting the overall survival rate of patients.

However, the above studies did not further explore the intra-operative and postoperative effects of different IMA types in patients undergoing laparoscopic radical resection of rectal cancer with LCA reservation.

### Effects of different IMA types on patients during and after the operation

Intra-operative blood loss can be used as an indicator of the success of laparoscopic surgery and can help surgeons determine the accuracy of vascular ligation [20]. In this study, we found that the amount of intra-operative blood loss in patients with type III IMA was higher than that in patients with type I and II IMA. This finding might be due to the fact that when the LCA, SA, and SRA were branched from the IMA at the same point, the three were close to each other and to the dense fibrous tissue around the IMA. Hence, the probability of accidental vascular injury was higher during LCA separation and ligation of SA and SRA. In addition, high ligation of the IMV at the lower margin of the pancreas is often required in laparoscopic LCA reservation surgery. However, Patroni [21] found that in IMA branch types, when the LCA, SA, and SRA branch from the IMA at the same point, the distance between the LCA at the lower margin of the pancreas and the IMV was less than the safe distance of surgery, which might also be the reason for more bleeding in type III IMA patients. Therefore, it was far from sufficient to only understand the classification of IMA before surgery. In patients with type III IMA, it is necessary also to understand the positional relationship between the IMA branches and adjacent vessels and nerves and measure the distance between the LCA and IMV at the lower margin of the pancreas and the IMA length, which are conducive to accurate clinical ligation.

The incidence of postoperative complications can be used as an indicator of patient prognosis. In this study, there was no statistical difference in the overall incidence of postoperative complications among the three groups. However, the incidence of anastomotic fistula in IMA patients with type III was higher than that in non-type III patients. This may be due to the low course of the type III IMA level 3 branch blood vessels (LCA, SA, SRA). When total mesorectal excision and pelvic floor anastomosis are performed, short LCA will also increase the tension on the suture line, resulting in vascular insufficiency and increasing the incidence of anastomotic leakage [22]. Huang [23] discussed the factors influencing postoperative anastomotic fistula in patients undergoing laparoscopic radical rectal surgery and found that type III IMA was an independent risk factor for anastomotic fistula. Therefore, for patients with type III IMA, intestinal recovery should be monitored, while abdominal signs should be recognized. Once an anastomotic fistula occurs, immediate treatment is required to prevent the formation of more serious complications, such as abdominal infection. In this study, we also analyzed the intestinal recovery function (first anal discharge time and retention time of abdominal drainage tube) and postoperative hospital stay of different patients and found no statistically significant differences.

### Limitations

This study has several limitations. First, the follow-up time was too short, and there was no further statistical analysis of the difference in long-term disease-free survival rate and overall survival rate among patients with different IMA types, which made it impossible to evaluate the overall prognosis for patients. Second, the other anatomical characteristics of IMA, such as IMA length, the position relationship between the IMA and IMV, and the influence of distance on the patients’ short-term efficacy, were not further analyzed in this study. Third, a small number of cases were included in this study, which might have led to deviations in the results. Therefore, large-sample, multicenter clinical studies are needed to provide evidence-based medical evidence.

## Conclusion

In conclusion, among patients undergoing LCA-preserving laparoscopic rectal cancer resection, patients with type III IMA had more intra-operative bleeding and a higher incidence of anastomotic fistula. However, in patients having type III IMA, the risk of overall postoperative complications did not increase. Before surgery, clinicians need to communicate with radiologists to understand the IMA branch types and the positional relationship of the surrounding blood vessels to deal with the blood vessels more accurately during surgery. Postoperative attention should be paid to the recovery of intestinal function in patients with type III IMA.

## Data Availability

The data that support the findings of this study are available on request from the corresponding author.

## References

[1] Heald R, Husband E, Ryall R (1982). The mesorectum in rectal cancer surgery—the clue to pelvic recurrence?. Br J Surg.

[2] Takahashi H, Haraguchi N, Nishimura J, Hata T, Matsuda C, Yamamoto H (2018). Laparoscopic lymph node dissection around the inferior mesenteric artery for left-sided colon and rectal cancer. Surg today.

[3] Tang B, Lei X, Ai J, Huang Z, Shi J, Li T (2021). Comparison of robotic and laparoscopic rectal cancer surgery: a meta-analysis of randomized controlled trials. World J Surg Oncol.

[4] Strhlein MA, Grützner KU, Jauch KW, Heiss MM (2008). Comparison of laparoscopic vs. open access surgery in patients with rectal cancer: a prospective analysis. Dis Colon Rectum.

[5] Abe T, Ujiie A, Taguchi Y, Satoh S, Shibuya T, Jun Y (2018). Anomalous inferior mesenteric artery supplying the ascending, transverse, descending, and sigmoid colons. Anat Sci Int.

[6] Liu X, Li J-b, Shi G, Guo R, Zhang R (2018). Systematic review of single-incision versus conventional multiport laparoscopic surgery for sigmoid colon and rectal cancer. World J Surg Oncol.

[7] Yada H, Sawai K, Taniguchi H, Hoshima M, Katoh M, Takahashi T (1997). Analysis of vascular anatomy and lymph node metastases warrants radical segmental bowel resection for colon cancer. World J Surg.

[8] Sakorafas GH, Zouros E, Peros G (2006). Applied vascular anatomy of the colon and rectum: clinical implications for the surgical oncologist. Surg Oncol.

[9] Wang K-X, Cheng Z-Q, Liu Z, Wang X-Y, Bi D-S (2018). Vascular anatomy of inferior mesenteric artery in laparoscopic radical resection with the preservation of left colic artery for rectal cancer. World J Gastroentro.

[10] Alici A, Kement M, Gezen C, Akın T, Vural S, Okkabaz N (2010). Apical lymph nodes at the root of the inferior mesenteric artery in distal colorectal cancer: an analysis of the risk of tumor involvement and the impact of high ligation on anastomotic integrity. Tech Coloproctol.

[11] Yang Y, Wang G, He J, Zhang J, Xi J, Wang F (2018). High tie versus low tie of the inferior mesenteric artery in colorectal cancer: a meta-analysis. Int J Surg.

[12] Girard E, Trilling B, Rabattu P-Y, Sage P-Y, Taton N, Robert Y (2019). Level of inferior mesenteric artery ligation in low rectal cancer surgery: high tie preferred over low tie. Tech Coloproctol.

[13] Fiori E, Crocetti D, Lamazza A, De Felice F, Scotti GB, Sterpetti AV (2020). Defecatory dysfunction after colon cancer resection: the role of inferior mesenteric artery tie. Anticancer res.

[14] Miles WE (1908). A method of performing abdomino-perineal excision for carcinoma of the rectum and of the terminal portion of the pelvic colon. The Lancet.

[15] Moynihan B (1908). The surgical treatment of cancer of the sigmoid flexure and rectum. Surg Gynecol Obstet.

[16] Maeda Y, Shinohara T, Futakawa N, Minagawa N, Sunahara M, Koyama R (2018). The oncologic outcomes of inferior mesenteric artery-preserving laparoscopic lymph node dissection for upper-rectal or sigmoid colon cancer. J Laparoendosc Adv S.

[17] Luo Y, Yu M-H, Huang Y-Z, Jing R, Qin J, Qin S-L (2021). Lymphadenectomy around inferior mesenteric artery in low-tie vs high-tie laparoscopic anterior resection: short-and long-term outcome of a cohort of 614 rectal cancers. Cancer Manag Res.

[18] You X, Liu Q, Wu J, Wang Y, Zhou Y (2020). High versus low ligation of inferior mesenteric artery during laparoscopic radical resection of rectal cancer: a retrospective cohort study. Medicine.

[19] Zhou J, Zhang S, Huang J, Huang P, Peng S, Lin J (2018). Accurate low ligation of inferior mesenteric artery and root lymph node dissection according to different vascular typing in laparoscopic radical resection of rectal cancer. Zhonghua Wei Chang Wai Ke Za Zhi.

[20] Kahnamoui K, Cadeddu M, Farrokhyar F, Anvari M (2007). Laparoscopic surgery for colon cancer: a systematic review. Can J Surg.

[21] Patroni A, Bonnet S, Bourillon C, Bruzzi M, Zinzindohoué F, Chevallier J (2016). Technical difficulties of left colic artery preservation during left colectomy for colon cancer. Surg Radiol Anat.

[22] Cirocchi R, Randolph J, Cheruiyot I, Davies JR, Wheeler J, Lancia M (2020). Systematic review and meta-analysis of the anatomical variants of the left colic artery[J]. Colorectal Dis.

[23] Huang J, Zhou J, Wan Y, Lin Y, Deng Y, Zhou Z (2016). Influences of inferior mesenteric artery types and Riolan artery arcade absence on the incidence of anastomotic leakage after laparoscopic resection of rectal cancer. Zhonghua Wei Chang Wai Ke Za Zhi.

